# Galanin mediates tumor-induced immunosuppression in head and neck squamous cell carcinoma

**DOI:** 10.1007/s13402-021-00631-y

**Published:** 2022-03-10

**Authors:** Marcell Costa de Medeiros, Min Liu, Rajat Banerjee, Emily Bellile, Nisha J. D’Silva, Carlos Rossa

**Affiliations:** 1 Department of Periodontics and Oral Medicine, School of Dentistry, University of Michigan, 1011 North University Ave, Room 2440, Ann Arbor, MI 48109-1078 USA; 2 Biostatistics, School of Public Health, University of Michigan, Ann Arbor, 48109 USA; 3 Department of Pathology, Medical School, University of Michigan, Ann Arbor, 48109 USA; 4 Rogel Cancer Center, University of Michigan, Ann Arbor, 48109 USA; 5 Department of Diagnosis and Surgery, School of Dentistry at Araraquara, UNESP- Univ Estadual Paulista, Rua Humaita 1680, Centro, Araraquara, SP CEP 14801-903 Brazil

**Keywords:** Head and neck cancer, Galanin, PBMCs, T lymphocytes

## Abstract

**Purpose:**

Galanin receptor 2 (GALR2) plays a significant role in the progression of head and neck squamous cell carcinomas (HNSCC). Since there is virtually no information on immunomodulation mediated by its ligand in the tumor microenvironment, we assessed the effects of galanin on peripheral blood mononuclear cells (PBMCs).

**Methods:**

After verification of GALR2 expression and it activity in PBMCs we evaluated the effect of galanin and conditioned media from HNSCC cell lines silenced for galanin or antibody-depleted, on proliferation, apoptosis, cytokine expression and activation/differentiation of immune cells.

**Results:**

We found that galanin alone and as a component of the HNSCC secretome decreased HNSCC cell proliferation and expression of pro-inflammatory cytokines (IFNγ, IL-12, IL-17A, IL-1α, IL-6 and TNF-α), whilst increasing apoptosis and expression of pro-tumoral cytokines/growth factors (IL-10, IL-4, PDGF and GM-CSF). T cell activation (using CD69 as activation marker) and anti-tumoral phenotypes in CD4^+^ T cells (Th1 and Th17) were found to be suppressed. In vivo, tumor growth was found to be increased in the presence of galanin-stimulated PBMCs. Data from The Cancer Genome Atlas (TCGA) revealed that high expression of galanin was associated with a reduced overall survival of patients with HNSCC.

**Conclusion:**

Our data indicate that galanin secreted by HNSCC cells exhibits immune-suppressive and pro-tumoral effects.

**Supplementary Information:**

The online version contains supplementary material available at 10.1007/s13402-021-00631-y.

## Introduction

Galanin has been implicated in multiple physiological and pathological processes in the nervous system, the endocrine system, metabolism, energy homeostasis and bone [1, 2]. Recent findings suggest a role for galanin in innate immunity, inflammation and cancer [1, 2]. Galanin induces three G protein coupled receptors: GALR1, GALR2 and GALR3 [3, 4]. GALR2 plays an important role in HNSCC [5–7] and stimulation of GALR2 activates RAP1, a small GTPase, that subsequently induces ERK, AKT and p38, leading to HNSCC cell proliferation, survival and the secretion of pro-angiogenic cytokines such as VEGF, respectively [5, 6]. In vivo, GALR2 induces invasion of HNSCC cells via NFATC2-mediated transcription of cyclooxygenase-2, which enzymatically facilitates prostaglandin E2 production, thereby promoting tumor progression [7]. In contrast, another study showed that GALR2 promotes apoptosis in HNSCC cells [8].

Galanin has been found to be constitutively secreted by various HNSCC cell lines [9]. A meta-analysis revealed that the expression of galanin was significantly higher in HNSCC tissues compared to normal (non-neoplastic) tissues [7]. Galanin and GALR2 mediate crosstalk between neural tissues and HNSCC cells, which ultimately induces neuritogenesis and facilitates perineural invasion [7]. Galanin plays an important role in perineural invasion, and there appears to exist a positive feedback loop (autocrine and paracrine) of GALR2 activation in the tumor microenvironment that stimulates and sustains tumor growth and invasion [7].

In late-stage HNSCC, total leukocyte counts are reduced compared to age-matched healthy individuals, and a decreased proliferative response of T lymphocytes from PBMCs has been associated with a worse prognosis [10]. The activation marker CD69 in T cells is reduced in HNSCC patients, and T-helper (CD4^+^) and cytotoxic T lymphocyte (CD8^+^) sub-populations have been found to be decreased in their peripheral blood [11]. Also, PBMCs from HNSCC patients produce lower levels of Th1-type cytokines (IFN-γ, IL-12 and TNF-α) and increased levels of Th2-type cytokines (IL-4 and IL-10) [11, 12]. Notably, an active immune response, characterized by increased prevalence of CD8^+^ T cells and Th1-type cells and cytokines, has been associated with a better prognosis [13, 14].

The recognition of crosstalk between neoplastic and immune cells has generated major breakthroughs in cancer biology and novel immunomodulatory therapeutic strategies [15]. Although this field of research has been rapidly expanding and shows therapeutic promise, in HNSCC the benefits of immunotherapy have been limited [16]. This may be due to a lack of profound understanding HNSCC tumor-immune interactions [16]. Various studies support an oncogenic role for GALR2 in HNSCC [5–7], suggesting an indirect influence of galanin as its ligand. The influence of galanin on the immune response is, however, largely unknown. It is conceivable that part of the increased aggressiveness of tumors showing higher secretion levels of galanin is mediated by immunomodulation. In fact, most studies report suppressive effects of galanin on immune cells. Galanin has been found to reduce the proliferation and increase the apoptosis of immature rat thymocytes [17] and to suppress the release of TNF-α by bacteria-stimulated murine macrophages [18].

Previously, we found that HNSCC-derived factors reduce the proliferation of immune cells, decrease the expression of pro-inflammatory cytokines (IL-12, IL-17A) and inhibit activation and polarization of CD4^+^ T cells to the Th17 phenotype [19]. These immunosuppressive effects may facilitate tumor evasion and actively co-opt immune cells to favor tumor cell proliferation, survival and migration. Considering the putative effect of galanin on HNSCC progression and a lack of information on possible immunomodulation mediated by tumor-derived galanin, we set out to investigate the effects of galanin on immune cells and to determine its relative contribution to the immunomodulatory effects of the HNSCC cell secretome. The identification of key soluble mediators derived from HNSCC cells may provide novel therapeutic targets for aggressive tumors and for rescuing anti-tumor immunity.

## Materials and methods

### Cells and culture conditions

HNSCC cell lines UM-SCC-1 and UM-SCC-22B [20, 21] were obtained from Dr. Thomas Carey, University of Michigan, USA. The cell lines were genotyped (University of Michigan DNA Sequencing Core) for authentication [20] and cultured in DMEM (11965–092; Gibco) supplemented with 10% FBS and 1% penicillin/streptomycin.

PBMCs were thawed according to the supplier’s instructions (Cellular Technologies) and used when the cell viability was over 90%, as determined by manual counting in a hemocytometer with 0.4% trypan blue. Non-stained cells were considered viable and counted as live cells. PBMCs from two male donors (derived from a single blood draw) were used in all experiments. Three independent experiments (biological replicates, two with one donor and one with the second), with 3 technical replicates in each independent experiment, were performed at 3 different times. Male donors were used due to the higher prevalence of HNSCCs in male patients [22].

### Proliferation and apoptosis assays

Proliferation was assessed at 24, 48, 72, 96 and 120 h. Apoptosis was determined at 96 h using an Annexin V/7-AAD assay according to the supplier’s instructions using a FACSVerse cytometer (BD Bioscience). Briefly, supernatants from non-attached and adherent cells dissociated for 10 min with enzyme-free dissociation buffer were combined and centrifuged (5 min, 400x *g*), after which apoptosis was quantified. Early and late apoptosis were considered in the apoptotic fraction (Annexin V^+^/7AAD^−^ and Annexin V^+^/7AAD^+^).

### GALR2 functionality assessment

PBMCs (1 × 10^6^/sample) were pre-treated with hrIL-2 (10 ng/ml) and Concavalin A (2.5 μg/ml) for 9 h prior to treatment with M871 (10 nM, 30 min) followed by 10 nM galanin.

### Conditioned medium collection

CM was collected from UM-SCC-1/UM-SCC-22B siNT and siGal cells. Briefly, cells were cultured in 100 mm dishes in complete DMEM until 60–70% confluence. Next, the cells were washed four times (2 h each) in 10 ml non-supplemented (‘blank’) RPMI-1640 and then incubated with 4 ml blank RPMI-1640 for 16 h. This medium was centrifuged (10,000 RPM, 10 min, 4 °C) to eliminate cell debris. The supernatant containing HNSCC-secreted products was designated CM, aliquoted immediately, frozen at -80 °C and used within 2 weeks.

### Depletion of galanin from CM

#### siRNA-mediated depletion

UM-SCC-1 and UM-SCC-22B cells were transfected with control non-targeting siRNA or siRNA targeting galanin using RNAiMAX and cultured for 48 h before initiation of CM production. Transient knockdown was confirmed by RT-qPCR and immunoblotting (Fig. 5B).

#### Antibody-mediated depletion

CM (4 ml for each 100 mm dish) generated from UM-SCC-1 and UM-SCC-22B cells as described above, were incubated with rabbit anti-galanin (1:200) or rabbit IgG (1:200, negative control), loaded onto 50 k Da filters and centrifuged (10,000 RPM, 10 min, 4 °C). To verify galanin depletion, aliquots of CM (pre-incubated with IgG or Gal-antibody) were analyzed by immunoblotting after concentration by centrifugation using 3 KDa filters (Millipore-Sigma).

### Effects of galanin and CM on immune cells

PBMCs were thawed and seeded at 1 × 10^4^ cells/well in 96-well plates for proliferation assays, and at 5 × 10^5^ cells/well (24-well plates; apoptosis) for flow cytometry. PBMCs were cultured with rhIL-2, concavalin A and phytohemagglutinin [23]. Immune cells were stimulated with galanin (10 or 300 nM) or CM from HNSCCs (or negative controls) in a proportion of 2:1 (vol:vol, CM:supplemented RPMI-1640). Proliferation was assessed by manual counting using trypan blue to exclude dead cells every 24 h, for 5 days. In the apoptosis assay, PBMCs were stimulated for 48 h, whereas for the activation (CD8) and phenotypic polarization assays, immune cells were stimulated for 96 h. We also evaluated the expression of spexin, which induces galanin receptor expression, in HNSCC cells, but its expression was very low compared with galanin and did not affect survival in TCGA datasets (Fig. S1).

### Immunoblotting

Concentrated CM and whole cell lysates prepared with lysis buffer were quantified using the Bradford method. Samples (20–30 μg) were electrophoresed in 4–12% gels under denaturing conditions (SDS-PAGE) and transferred to 0.2 μm nitrocellulose membranes. The antibodies used are listed in Table S1.

### RT-qPCR

Cells were lysed with QIAzol after which total RNA was extracted using an affinity column system, which includes treatment with DNase. Next, cDNA was synthesized from total RNA (500 ng) using random hexamers as primers and a commercially available system. Real time PCR was performed using TaqMan chemistry and pre-designed and optimized Taqman Gene Expression Assays. All mRNA expression levels are presented as 2^-(GoI-RG).

### Multiplex analysis of PBMCs-secreted products

PBMCs (2 × 10^6^) were cultured in the absence (PBS Control) or presence of galanin (10 and 300 nM) and  supernatants were collected after 96 h. Cytokines/chemokines/growth factors were quantified by Luminex assay on a Bio-plex200 system (Bio-Rad) following the manufacturer’s protocol. Replicates from each of three independent experiments were combined to generate data for each group for analysis (each heat map square represents a pool of three replicates). Cytokines/chemokines/growth factors included in the heat map exhibited at least a 50% change at any galanin concentration (fold change < 0.5 or > 1.5) after normalization to control (no galanin).

### Flow cytometry; phenotype and activation of immune cells

Cells were collected after 96 h stimulation with vehicle (PBS Control), 10 nM or 300 nM galanin. Galanin was only added at 0 h after which the culture medium was unchanged until collection. Fc receptors were blocked and the cells were subsequently stained with fluorescent-conjugated primary antibodies at optimized dilutions. For staining of intracellular cytokines or FOXP3, after washing in PBS, cells were fixed in 4% paraformaldehyde and permeabilized with saponin-containing buffer. Activation of CD3^+^ and CD8^+^ cells was assessed by CD69 expression. The phenotype of CD4 cells was studied by co-expression of CD4 and IFNγ (Th1), IL4 (Th2) or IL17A (Th17). Tregs were detected by co-expression of CD4, FOXP3 and CD25. A minimum of 10,000 events (after exclusion of cell debris and doublets) were acquired using a FACSVerse flow cytometer and analyzed using FACSuite software (BD Biosciences).

### Chick chorioallantoic membrane (CAM) assay

CAM assays were used to evaluate tumor growth [24]. UM-SCC-29 cells, stained with green cell tracker (Molecular Probes) for 1 h and pre-treated PBMCs were grafted for 72 h on CAMs [25]. The PBMCs were pre-treated for 96 h before grafting with: (i) activation cocktail (rhIL-2, CD3/CD28 activation beads and LPS) and (ii) activation cocktail plus galanin (10 nM). At grafting, fresh PBMCs were thawed and used as non-activated group. Experimental groups were: 1) UM-SCC-29 cells, 2) non-activated PBMCs + UM-SCC-29 cells, 3) activated PBMCs + UM-SCC-29 cells and 4) activated PBMCs + galanin + UM-SCC-29 cells. Integrated densities of fluorescence images were used to measure tumor areas using image J software (https://imagej.nih.gov/ij/ - Rasband, W.S., ImageJ, U. S. National Institutes of Health, Bethesda, MA, USA, 1997-2018).

### Datasets

RNA expression data from 529 primary HNSCCs were obtained from the TCGA database (https://tcga-data.nci.nih.gov/tcga/). Information from HNSCC patients, including galanin and CD8 mRNA expression, survival status and survival duration in years, were downloaded from UCSC Xena for Cancer Genomics (https://xena.ucsc.edu).

### Data analysis

**Fig. 1:**
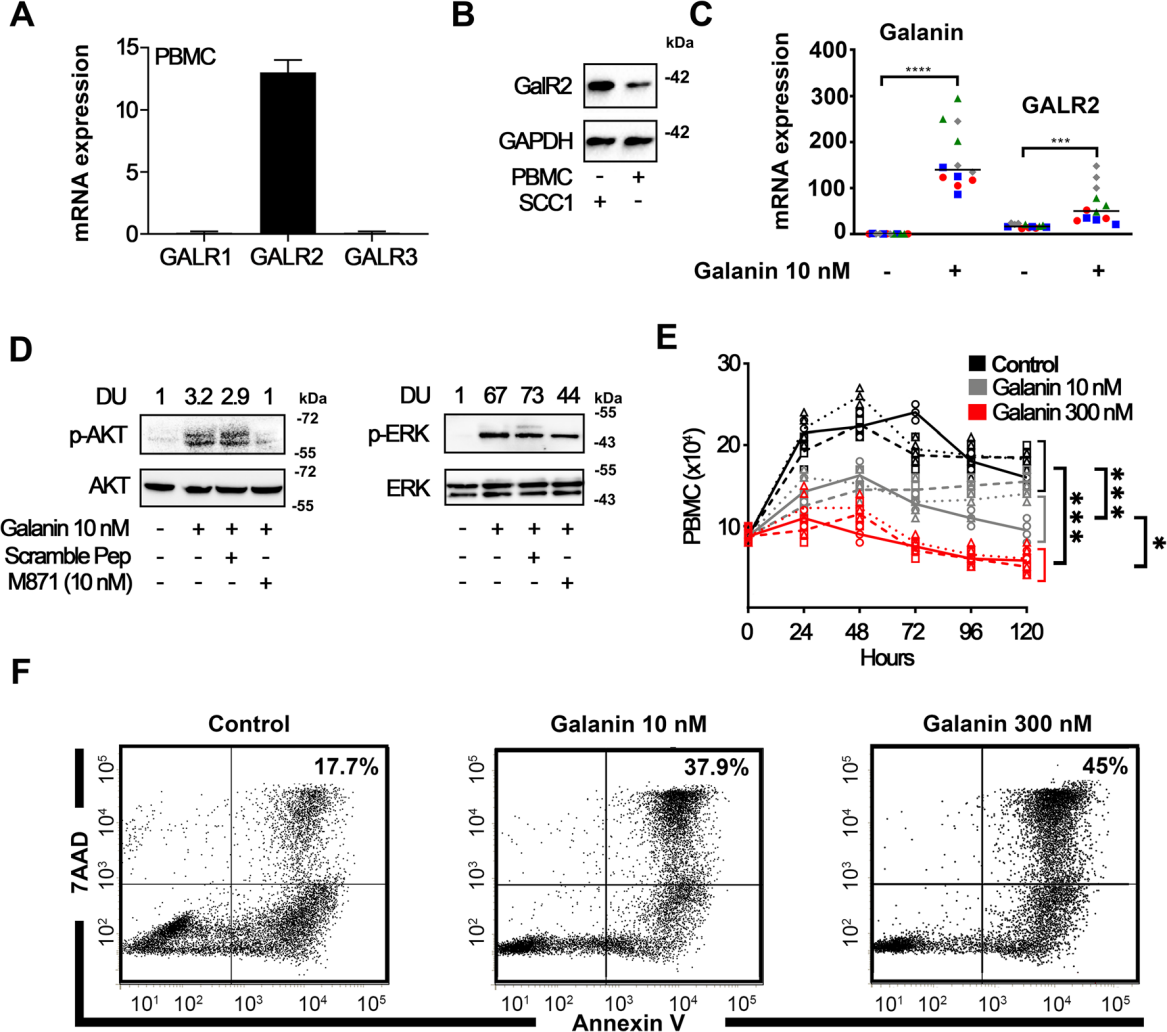
**Galanin inhibits immune cell proliferation via GALR2.** Quantification of mRNA expression of GALR1, GALR2 and GALR3 in PBMCs (**A**), and expression of GALR2 confirmed by immunoblotting (UM-SCC-1: positive control) (**B**). Data are representative of 2 independent experiments with 2 different donors. (**C**) Gene expression of galanin and GALR2 in PBMCs after 24 h stimulation with 10 nM galanin. (**D**) Functionality of GALR2 on PBMCs. AKT and ERK phosphorylation induced by galanin is reduced by M871, a GALR2 antagonist. (**E**) Galanin inhibits proliferation of PBMCs. Different line styles represent each independent experiment; two independent experiments with PBMCs of donor #1 and a third independent experiment with PBMCs of donor #2 were performed. (**F**) Apoptosis (Annexin^+^/7AAD^+^ combined) of PBMCs. Each independent experiment encompassed three replicates. * *p* < 0.05, * *p* < 0.01, *** *p* < 0.001 and **** *p* < 0.0001

GraphPad Prism (v8 for Windows, GraphPad Software, CA, USA) and SAS (v9.4, SAS Institute, NC, USA) were used for analyses. The purpose of the analyses was to determine the significance of galanin (either as an independent stimulus or as a component of HNSCC cell-secreted products) on the outcomes of interest. The effects of galanin were compared to a single appropriate negative control for each HNSCC cell line. There was no intent to compare outcomes between different HNSCC cell lines or donor PBMCs. Technical replicates displayed in figures were averaged for analyses. One-way ANOVA with post-hoc Tukey tests were used for multiple comparisons among the experimental conditions (Control, Galanin 10 nM and Galanin 300 nM). Student’s t test was applied for two group comparisons (siNT versus siGal and IgG versus AbGal). For experiments with measurements over time (proliferation assay, Fig. 1C), a linear mixed effects model (LMM) was used. Each model included categorical fixed effects for hour, group and hour*group interactions, and appropriate contrasts were used for testing differences between groups at each time point. The LMM assumed within-experiment correlation with an autoregressive correlation structure. Survival probability curves were calculated using the Kaplan–Meier method and tested by log-rank test. Spearman correlation test was used to investigate associations between CD8 and galanin. Significance levels of *p* < 0.05, *p* < 0.01, *p* < 0.001 and *p* < 0.0001 are annotated with one, two, three and four asterisks, respectively.

## Results

### Immunomodulatory effects of galanin 

The expression of galanin receptors by PBMCs was verified by RT-qPCR and immunoblotting. GALR2 was the only galanin receptor expressed by immune cells and was upregulated after direct stimulation with galanin (Fig. 1A-C). GALR2 is functional in PBMCs since 10 nM galanin activated AKT and ERK, whereas M871, a GALR2-specific inhibitor [26], reduced this activation (Fig. 1D). These results indicate that these pathways are linked to galanin/GALR2 signaling in immune cells. Since the effects of galanin may be concentration-related, we determined the effects of two concentrations of galanin (10 and 300 nM). In doing so, we observed a concentration-dependent decrease in mitogen-induced proliferation of PBMCs after 96 h (Fig. 1E), as well as an increase in apoptosis (Fig. 1F). Collectively, these data indicate that PBMCs express functional GALR2 and that galanin dose-dependently reduces proliferation and increases apoptosis of these cells.

**Fig. 2:**
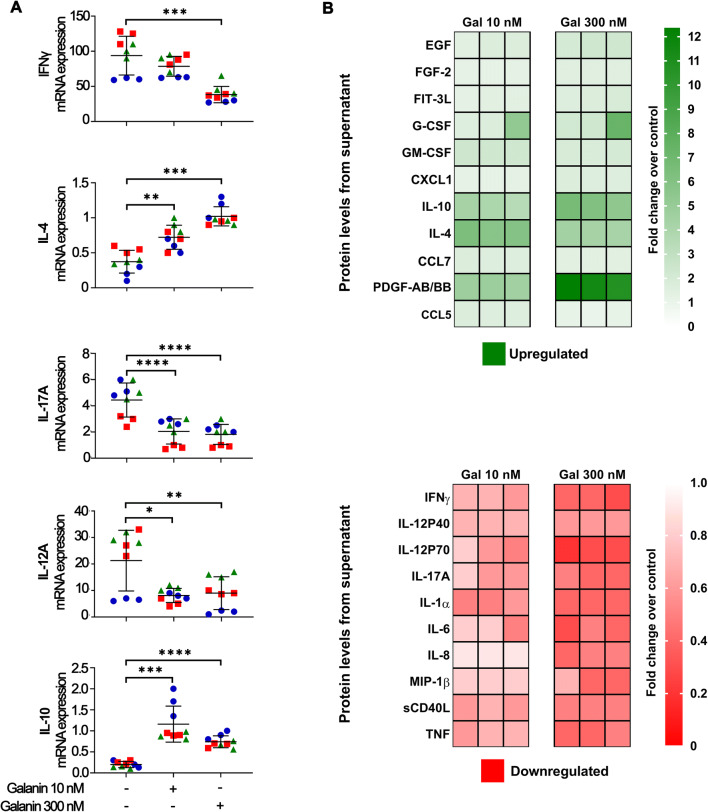
**Galanin shifts the PBMC cytokine profile to suppressive/pro-tumoral**. (**A**) mRNA cytokine expression signatures of anti-tumoral (IFNγ, IL-12A, IL-17A) and pro-tumoral (IL-4, IL-10) phenotypes. (**B**) Heat map of the fold changes (FC) of cytokines, chemokines and growth factors secreted by PBMCs evaluated by multiplex assay. Green shades indicate upregulated (FC > 1.5) and red shades indicate downregulated (FC < 0.5) markers. Overall, anti-tumoral cytokines such as IFNγ, IL-17A, IL-12A, IL-1α and TNF were downregulated in the presence of galanin. Also, pro-tumoral IL-4 and IL-10 cytokines were upregulated. Panel A: each color/shape represents an independent experiment. Two independent experiments with PBMCs of donor #1 and a third independent experiment with PBMCs of donor #2 were performed. Each independent experiment encompassed three replicates. ** *p* < 0.01, *** *p* < 0.001 and **** *p* < 0.0001

The effects of galanin on the immune cell phenotype was assessed by gene expression analysis of T helper (IFNγ, IL-4, IL-17A) and macrophage (IL-12, IL-10) specific cytokines. Comprehensive assessment of galanin in immune cell-secreted products was carried out using a multiplex assay. We found that galanin suppressed pro-inflammatory (IFNγ, IL-17A, IL-12A) and increased anti-inflammatory (IL-4, IL-10) cytokines (Fig. 2A). Moreover, although not statistically significant, there was a trend towards a concentration-dependent increase in IL-4 and decrease in IFNγ, further indicating a Th2-skewing effect of galanin. Galanin increased the secretion of growth factors such as EGF, FGF-2, and especially PDGF-AB/BB, G-CSF, GM-CSF and FLT3L and chemokines (CXCL1, CCL7, CCL5) (Fig. 2B), whereas MIP-1β and IL-8 were markedly downregulated by galanin. There was an increase in cytokines associated with pro-tumoral effects (e.g., IL-10, IL-4) and a decrease in those associated with anti-tumoral effects (TNF, IFNγ, IL-6, IL-12, IL-17) (Fig. 2B). For some of these cytokines, a concentration-dependent effect was observed with more pronounced changes (increase/decrease) being associated with 300 nM rather than 10 nM galanin (e.g., EGF, FLT3L, G-CSF, IL10, IL12, PDGF). Collectively, these data suggest that galanin suppresses immune cells.

**Fig. 3:**
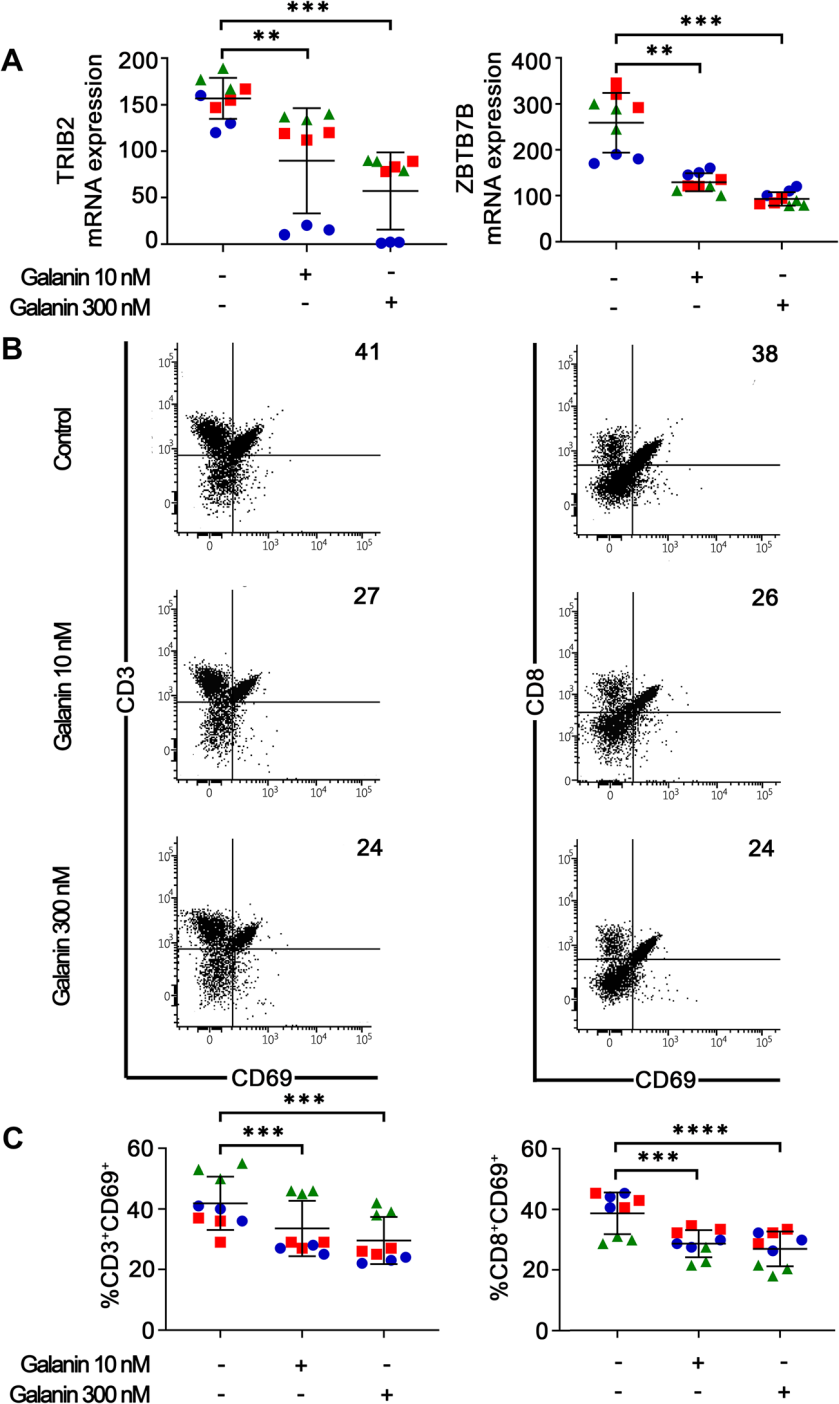
**Galanin reduces T cell activation.** (**A**) Galanin reduced mRNA expression of TRIB2 and ZBTB7B in PBMCs after 96 h. These markers are linked to activation and maturation of CD3^+^ to CD4+ or CD8^+^ lineages, respectively. (**B**) Representative dot-plots of flow cytometry assessment of the effects of galanin on CD69 expression (a marker of early activation) by CD3^+^ (left) and CD8^+^ (right) T cells. Lower graphs represent data from all independent experiments showing a reduction in active subpopulations (CD3^+^CD69^+^ and CD8^+^CD69^+^). Each color/shape represents an independent experiment. Two independent experiments with PBMCs of donor #1 and a third independent experiment with PBMCs of donor #2 were performed. Each independent experiment encompassed three replicates. ** *p* < 0.01, *** *p* < 0.001 and **** *p* < 0.0001

Galanin suppressed the mRNA levels of ZBTB7B and TRIB-2 (Fig. 3A), which are known to be responsible for CD3 maturation (toward CD4 or CD8) and for MAPK signaling associated with cell activation, respectively [27, 28]. Expression of the activation marker CD69 by CD3^+^ and CD8^+^ T cells was also found to be reduced by galanin (Fig. 3B, C). No concentration-dependent effect was observed. These results support the notion that galanin reduces activation of CD4^+^ and CD8^+^ T cells.

**Fig. 4:**
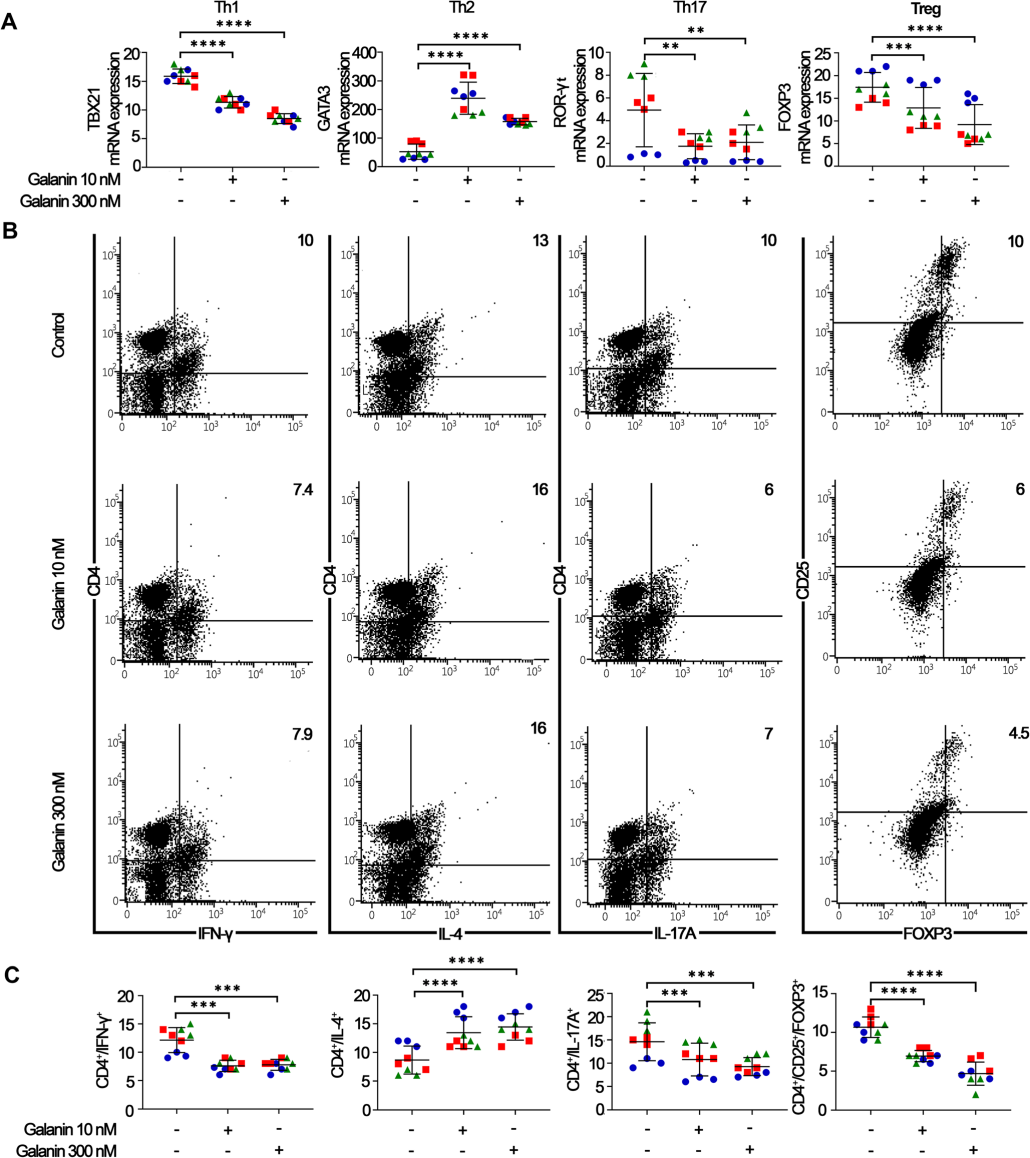
**Galanin induces immunosuppressive skewing of T helper-type phenotypes.** (**A**) Regulation of gene expression of T helper-type transcription factors associated with Th1 (Tbet), Th2 (GATA3), Th17 (RORγt) and Treg (FOXP3) by galanin stimulation in PBMCs. (**B**) Representative dot-plots of the effect of galanin stimulation on the immunophenotype of T helper (CD4^+^) cells. (**C**) Graphs presenting data from all independent experiments depicting the effect of galanin stimulation on the immunophenotype of T helper (CD4^+^) cells. Different colors/shapes represent independent experiments. Two independent experiments with PBMCs donor #1 and a third independent experiment with PBMCs donor #2 were performed. Each independent experiment encompassed three replicates. **p < 0.01, ***p < 0.001 and ****p < 0.0001

HNSCCs have an immunosuppressive profile [29, 30]. CD4^+^ T cells orchestrate pro- and anti-inflammatory states through phenotypic plasticity. In this context, the effect of galanin on the phenotypic polarization of CD4^+^ T cells was investigated. PBMCs were stimulated with galanin (96 h, 10 nM and 300 nM) after which the expression of T helper phenotype-associated transcription factors and cytokine markers were studied by RT-qPCR and flow cytometry, respectively. At both concentrations, galanin polarized CD4^+^ T cells to a predominantly anti-inflammatory phenotype, as the expression of T-bet (Th1-related) and RORγt (Th17-related) transcription factors was reduced, and GATA3 gene expression (Th2-related) was increased (Fig. 4A). Flow cytometry analysis supported these findings, as galanin stimulation reduced the proportion of Th1 (CD4^+^/IFNγ^+^) and Th17 (CD4^+^/IL17A^+^) cells and increased the proportion of Th2 (CD4^+^/IL4^+^) cells (Fig. 4B, C). Of note, galanin also reduced the proportion of Tregs (CD4^+^/CD25^+^/FOXP3^+^). Taken together these findings indicate that galanin skews CD4^+^ T cells to anti-inflammatory/pro-tumoral phenotypes.

### Effects of galanin as component of the HNSCC cell secretome

**Fig. 5:**
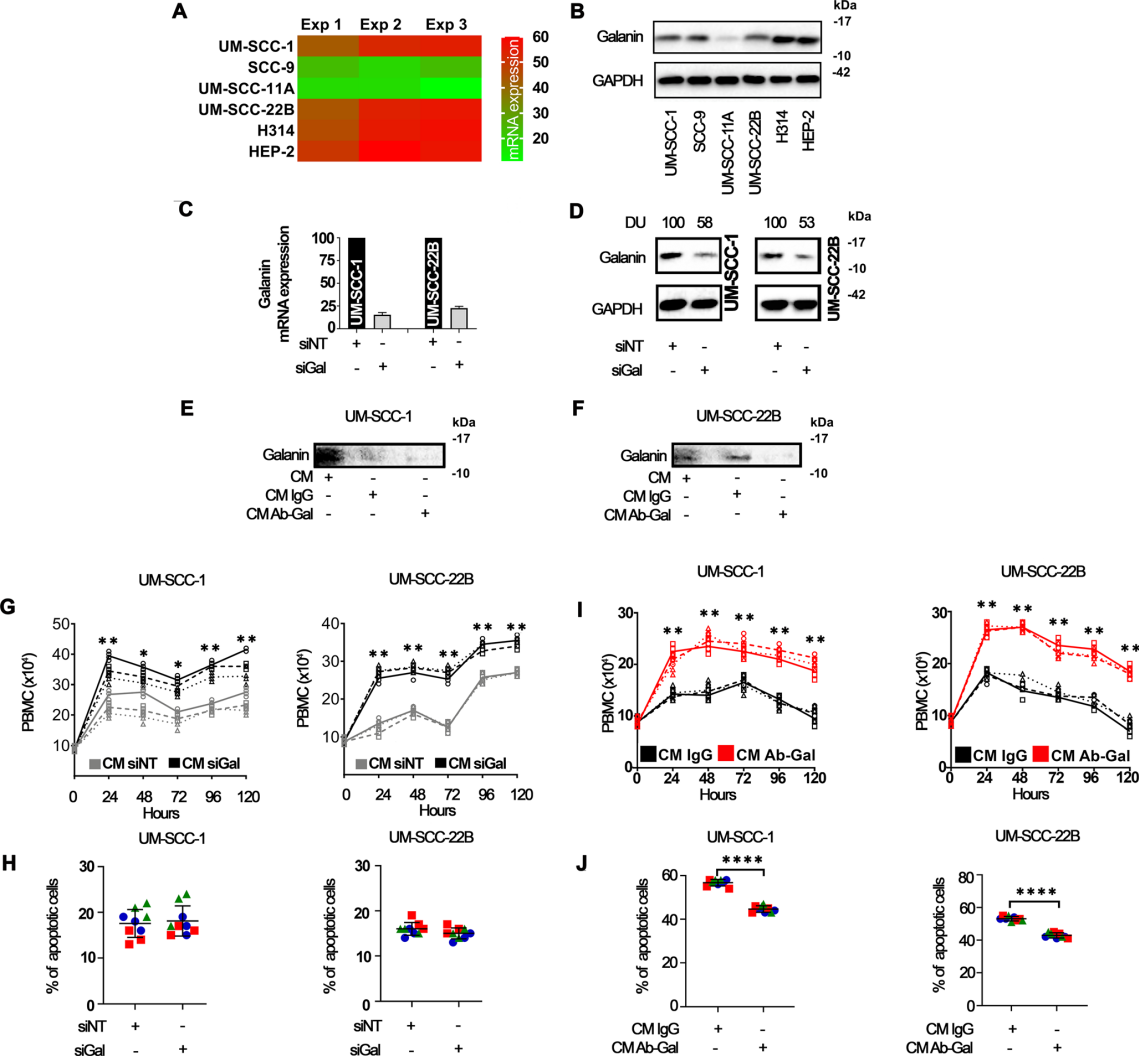
**HNSCC-secreted galanin reduces the proliferation of immune cells.** Constitutive expression of galanin mRNA (**A**) and protein (**B**) in a panel of HNSCC cells. (**C**) and (**D**): Validation of siRNA-mediated silencing of galanin in UM-SCC-1 and 22B cells. (**E** and **F**): Efficacy of antibody-mediated depletion of galanin from the CM of HNSCC cells. (**G**): Increased proliferation of immune cells exposed to CM from siGAL HNSCC cells, without significant reduction in apoptosis with CM (**H**). Exposure to galanin-depleted CM significantly increased immune cell proliferation (**I**) and reduced apoptosis (**J**). Different line styles represents independent experiments. Two independent experiments with PBMCs of donor #1 and a third independent experiment with PBMCs of donor #2 were performed. Each independent experiment encompassed three replicates. ** *p* < 0.01, *** *p* < 0.001 and **** *p* < 0.0001

Using RT-qPCR and immunoblotting we found that galanin expression was heterogenous in a panel of HNSCC cell lines (Fig. 5A, B). UM-SCC-1 and UM-SCC-22B cells were selected for subsequent experiments. Two orthogonal approaches determined the impact of HNSCC-secreted galanin on PBMCs using siRNA- (Fig. 5C, D) and antibody-mediated (Fig. 5E, F) depletion. We found that siGAL decreased galanin protein levels to 58% and 53% of siNT in UM-SCC-1 and UM-SCC-22B cells, respectively (Fig. 5D), and that galanin was depleted by 81% in CM from UM-SCC-1 cells (Fig. 5E) and 90% in CM from UM-SCC-22B cells (Fig. 5F). CM from cells transfected with siGAL significantly enhanced IL-2/mitogen-induced proliferation of PBMCs (Fig. 5G), but did not affect their apoptosis (Fig. 5H). Antibody-mediated depletion of galanin in CM (Fig. 5E, F) increased the proliferation of PBMCs (CM ab-Gal) compared to control (CM IgG) (Fig. 5I) and decreased their apoptosis (Fig. 5J). These results support the notion that HNSCC-secreted galanin suppresses the proliferation of PBMCs.

**Fig. 6:**
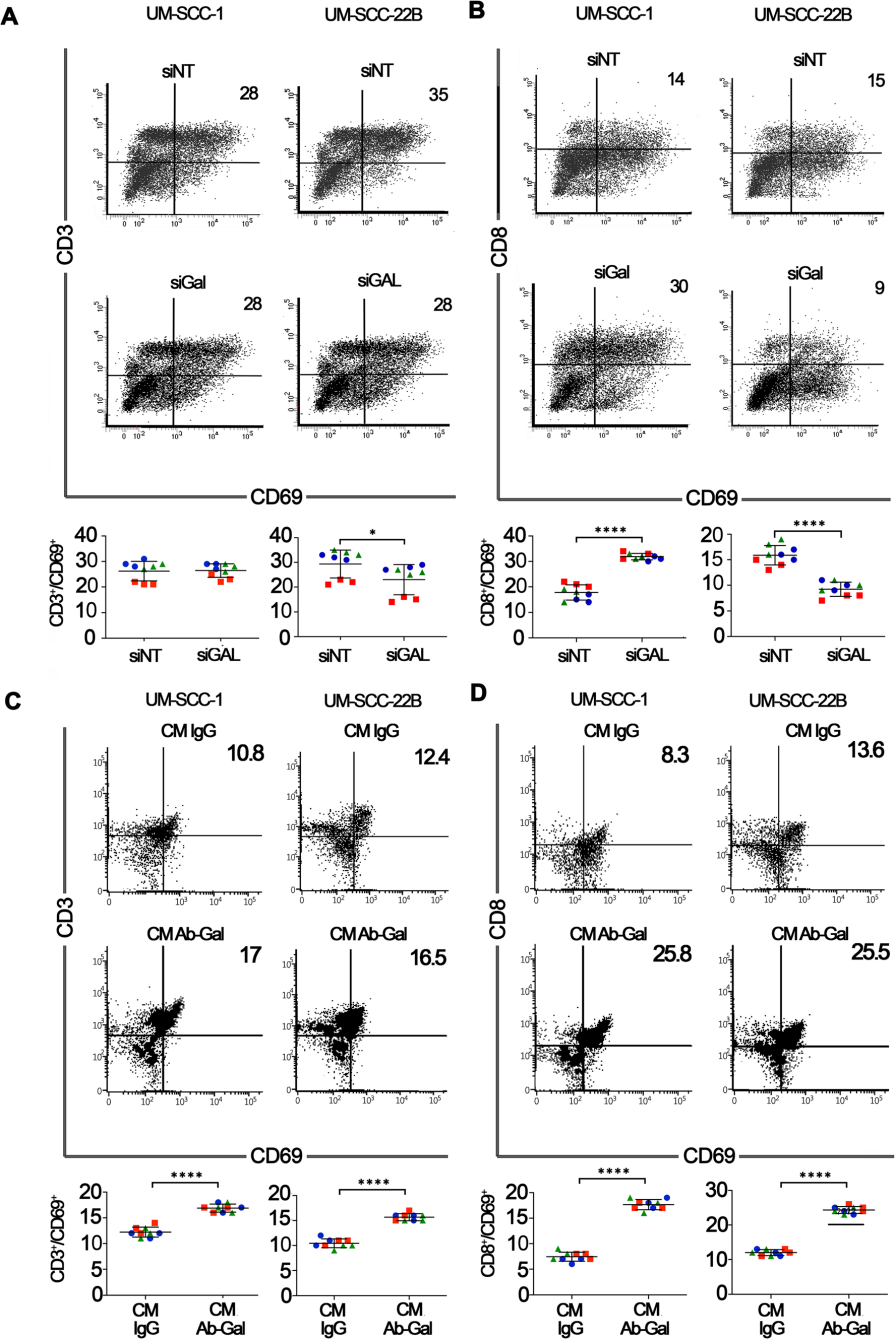
**HNSCC-secreted galanin modulates T cell activation.** Activation of CD3^+^ (**A**) and CD8^+^ (**B**) cells (CD3^+^/CD69^+^ and CD8^+^/CD69^+^, respectively) after treatment of PBMCs with CM from UM-SCC-(1 and 22B) with siRNA-mediated silencing of galanin. UM-SCC1-secreted galanin did not affect activation of CD3^+^ cells, but increased activation of CD8^+^ cells. UM-SCC-22B-secreted galanin reduced activation of both CD3^+^ and CD8^+^ cells. A complementary experimental approach of antibody-mediated depletion indicates that tumor cell-secreted galanin significantly inhibited activation of both CD3^+^ (**C**) and CD8^+^ (**D**) cells. In all graphs, each color/shape represents an independent experiment with three replicates in each experiment. Different colors/shapes between the same group represent independent experiments. Two independent experiments with PBMCs of donor #1 and a third independent experiment with PBMCs of donor #2 were performed. Each independent experiment encompassed three replicates. * *p* < 0.05 and **** *p* < 0.0001

HNSCC-secreted galanin exhibited differential effects on the activation of CD3^+^ and CD8^+^ T cells depending on the HNSCC cell line and experimental approach used. Silencing of galanin in UM-SCC-1 cells increased activation of CD8^+^ but not CD3^+^ cells, whereas CM from galanin-silenced UM-SCC-22B cells reduced activation of both CD3^+^ and CD8^+^ T cells (Fig. 6A, B). In contrast, we found that galanin depletion from the CM of both UM-SCC-1 and UM-SCC-22B cells increased the activation of CD3^+^ and CD8^+^ T cells in comparison with control (IgG) CM (Fig. 6C, D). Of note, consistent with the inhibition of T cell activation by galanin as an independent stimulus (Fig. 3), we found that reduction of galanin in the secretome of HNSCC cell lines alleviated the suppression of CD3^+^ and CD8^+^ T cells.

**Fig. 7:**
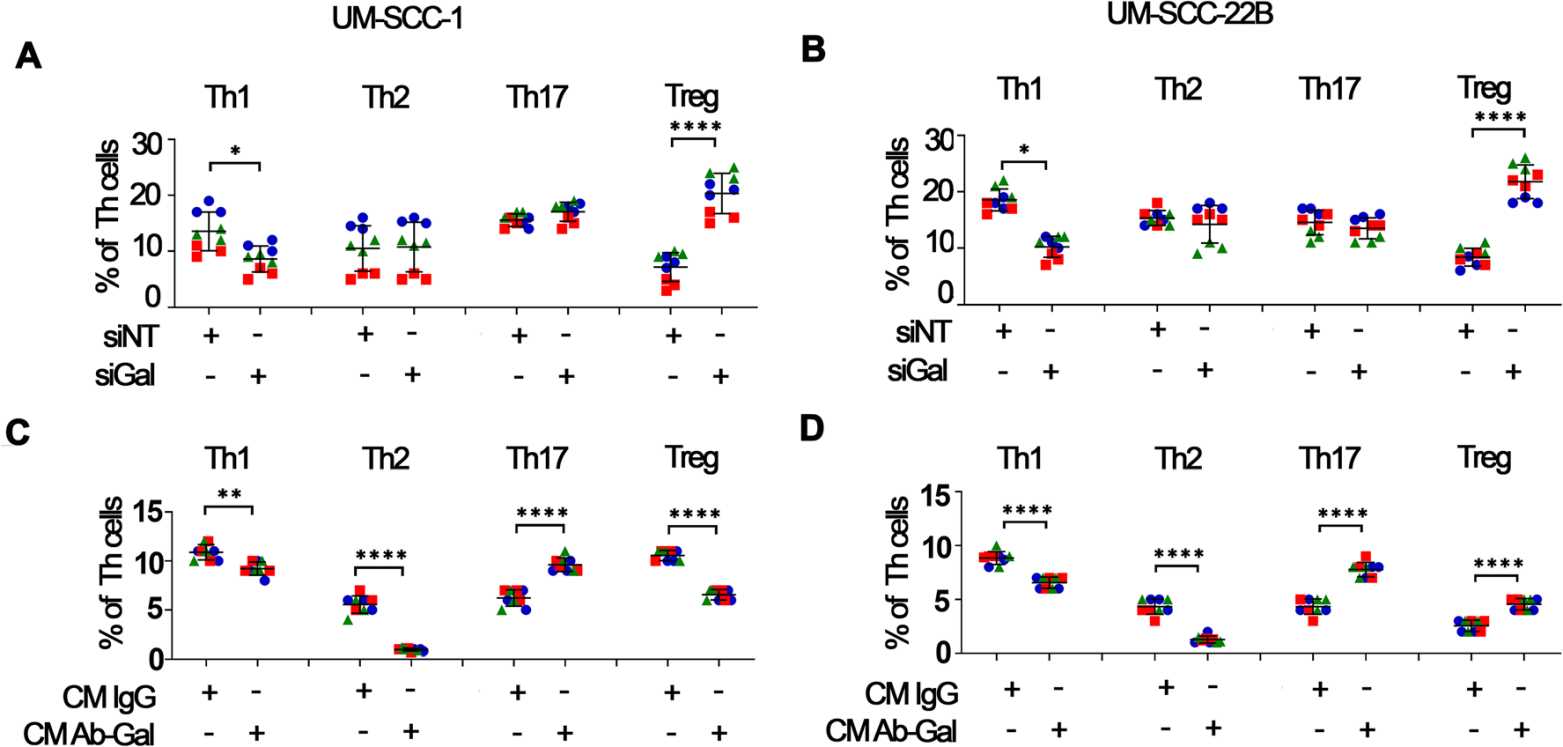
**HNSCC-secreted galanin suppresses the T helper-type response.** Exposure of PBMCs to secreted products of HNSCC cells with siRNA-mediated silencing of galanin markedly reduced the proportion of Th1 cells and increased the proportion of Tregs, whereas the proportions of Th17 and Th2 cells were not affected (**A** and **B**). Exposure of PBMCs to secreted products of HNSCC cells with antibody-mediated depletion of galanin (**C** and **D**) reduced the proportions of Th1 and Th2 cells, while the proportion of Th17 cells was increased. The proportion of Tregs was differentially affected according to the HNSCC cell line used: decreased upon exposure to galanin-depleted CM from UM-SCC-1 and increased upon exposure to galanin-depleted CM from UM-SCC-22B. The magnitude of changes in the proportion of cells in each phenotype indicates suppressive effects of galanin as a component of HNSCC cell-secreted products. Different colors/shapes between the same group represent independent experiments. Two independent experiments with PBMCs of donor #1 and a third independent experiment with PBMCs of donor #2 were performed. Each independent experiment encompassed three replicates. * *p* < 0.05, ** *p* < 0.01 and **** *p* < 0.0001

Next we determined the impact of HNSCC-secreted galanin on the phenotype of CD4^+^ T cells. We found that silencing of galanin in UM-SCC-1 and UM-SCC-22B cells resulted in consistent effects on the CD4^+^ phenotype: reductions in Th1 cells and increases of Tregs, whereas Th2 and Th17 cells remained unchanged (Fig. 7A, B). Antibody-mediated depletion of galanin from CM of both HNSCC cell lines also reduced the proportion of Th1 cells. However, in contrast with the siRNA approach, we observed a marked decrease in the proportion of Th2 cells and an increase in the proportion of Th17 cells (Fig. 7C, D). Moreover, depletion of galanin from CM of UM-SCC-1 cells reduced the proportion of Tregs, whereas galanin-depleted CM from UM-SCC-22B cells markedly increased the proportion of Tregs (Fig. 7C, D).

The immunosuppressive effects of tumor-derived galanin varied according to the experimental approach used to reduce galanin. CM from galanin-silenced UM-SCC-1 cells only inhibited the activation of CD8^+^ T cells, whereas with the antibody-depletion approach, galanin-depleted CM from both UM-SCC-1 and UM-SCC-22B cells enhanced the activation of both CD3^+^ and CD8^+^ T cells. Besides differences in efficacy of these two approaches in reducing galanin in the CM, it is possible that the siRNA approach prevented autocrine/paracrine effects of galanin, which may have influenced the production of other soluble mediators. In the antibody-mediated depletion approach, secreted galanin may have exerted these autocrine/paracrine effects prior to removal. Additionally, off-target effects associated with the siRNA approach may have influenced the HNSCC secretome.

### Influence of immunomodulatory effects of galanin in vivo and correlation with patient data

**Fig. 8:**
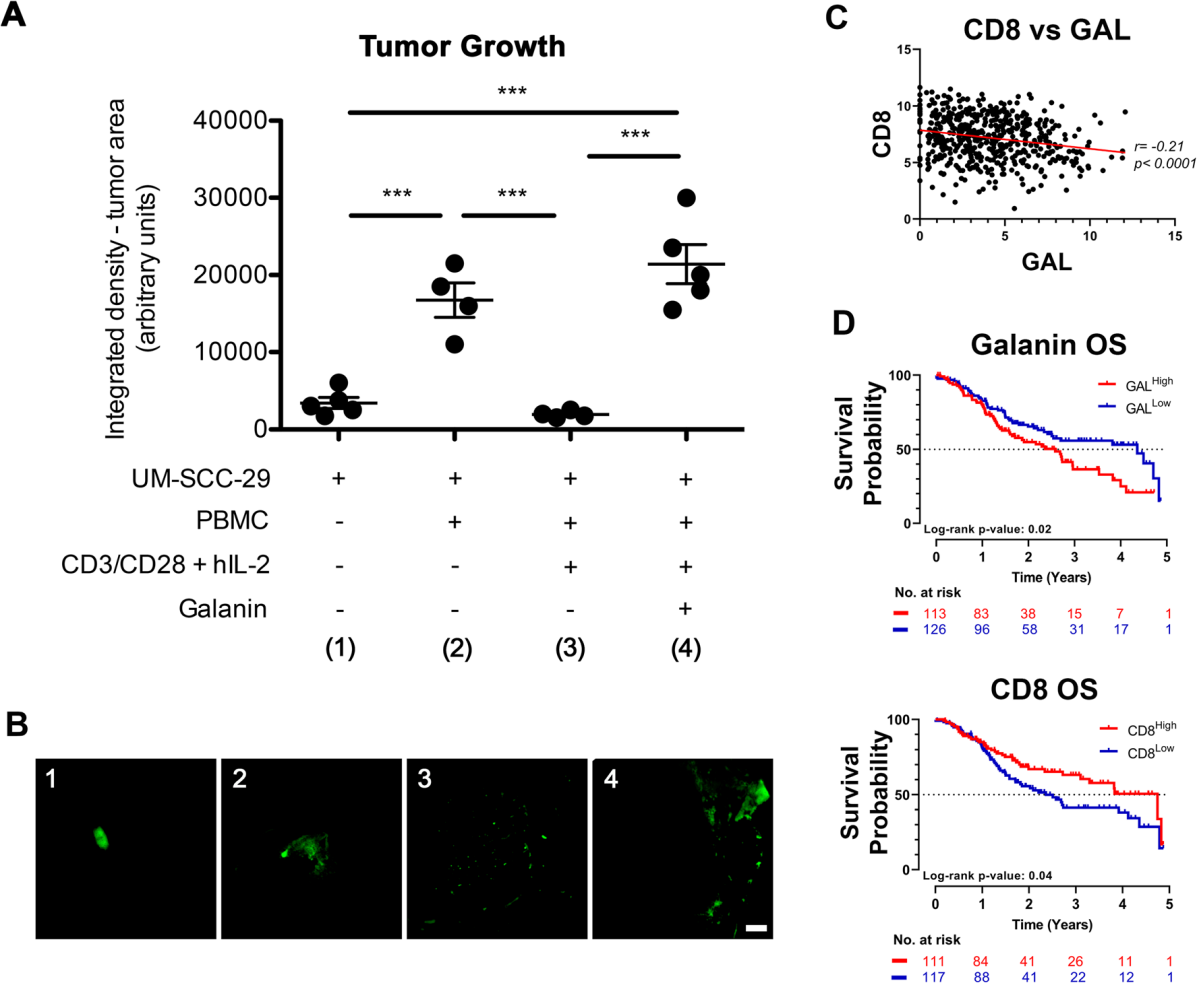
**Galanin-induced immunosuppression favors tumor growth in vivo; galanin is inversely correlated with CD8 and high levels of galanin are associated with a worse survival in patients with HNSCC.** PBMCs were co-cultured with UM-SCC-29 cells under different pre-treatment conditions. Tumor growth was measured by integrated density from fluorescent images. (**A**) Galanin pre-treatment of PMBCs allowed a larger tumor growth compared to activated PBMCs. (**B**) Representative images of tumors at each condition. (**C**) TCGA data showing that CD8 and galanin expression are negatively associated. (**D**) Overall survival (OS) data from the TCGA dataset correlating high and low levels of galanin and CD8. Low galanin and high CD8 mRNA levels are associated with a better survival probability. Scale white bar represents 1 mm. *** *p* < 0.001

To evaluate the immunomodulatory effects of galanin on tumor growth in vivo, PBMCs and UM-SCC-29 cells were co-grafted on CAMs. We found that activated (CD3/CD28 + hIL-2 cocktail) PBMCs, primed with galanin, significantly enhanced tumor growth compared to UM-SCC-29 cells alone or UM-SCC-29 cells co-grafted with activated PBMCs (Fig. 8A, B), supporting the notion that immunomodulation by galanin favors tumor growth. In TCGA data from HNSCC patients, galanin and CD8 showed an inverse correlation (r = −0.21, *p* < 0.0001) (Fig. 8C). Survival plots of these patients showed a worse survival with high levels of galanin compared to those with low levels, and patients with high levels of CD8 showed a better 5-year survival probability (Fig. 8D).

## Discussion

A prominent suppressive effect of HNSCC cells on the immune microenvironment [31] has been highlighted by the efficacy of immune checkpoint inhibitors [32–34]. However, less than 20% of patients with HNSCC benefit from these inhibitors, emphasizing the importance of identifying additional mechanisms of immune suppression for new therapeutic strategies. In this study, we assessed the immunomodulatory effects of galanin in vitro and in vivo in HNSCC.

Overall, we found that galanin alone had an immunosuppressive effect on PBMCs, as indicated by decreased IL-2/mitogen-induced proliferation, suppression of pro-inflammatory and upregulation of anti-inflammatory mediators, inhibition of CD3^+^ and CD8^+^ T cells, and skewing of CD4^+^ T cells to anti-inflammatory/pro-tumoral phenotypes. These immunosuppressive effects are consistent with other reports [17, 18, 35], but in contrast with studies that reported an immunostimulatory effect [36], or both effects depending on microenvironmental conditions [37]. We speculate that these discrepancies may be related to the experimental context (in vitro versus in vivo) and conditions (cell type, galanin concentration, duration of stimulation). We used two concentrations of galanin, which yielded overall similar results except for discrete concentration-dependent effects on proliferation/apoptosis and on regulation of cytokine expression/production. Differential expression of GALR1, GALR2 and GALR3 by different cell types may also account for contrasting biological effects [3]. Interestingly, recent evidence indicates that the expression of galanin, GALR1 and GALR2 may vary with the differentiation and phenotype of macrophages (M0, M1 or M2) and may influence the production of anti-inflammatory cytokines (TGF- β, IL-10 and IL-1Ra) in M1 macrophages [37].

Here, PBMCs were used to study the immunomodulatory effects of galanin, maintaining interactions of different immune cell types. We found that only GALR2 was expressed by PBMCs, which is consistent with the reported expression of only GALR2 in NK cells [36] and neutrophils [1]. Although GALR1 and GALR3 expression by PBMCs has been reported, this discrepancy may be related to the genetic/phenotypic background of the PBMC donors and with the experimental approaches (Northern blotting versus RT-qPCR) used [38, 39]. Importantly, expression (mRNA and protein) and functionality of GALR2 in PBMCs were both determined in our current study, which supports our interpretation that the immunomodulatory effects of galanin result from activation of GALR2.

Previous studies have shown that GALR2 expression increases the progression of HNSCC, and that its activation results in a positive autocrine regulatory loop that increases secretion of its ligand, galanin [5, 7]. Moreover, the galanin/GALR2 signaling axis has been reported to be involved in crosstalk between neoplastic and neuronal cells, leading to perineural invasion (PNI) and increased HNSCC invasion [40]. It is tempting to speculate that the immunosuppressive effects of galanin via GALR2 may represent another example of crosstalk between neoplastic/neuronal/immune cells in the tumor microenvironment that facilitates immune evasion and even co-opts immune cells to enhance tumor growth and invasion.

To obtain insight into the relative contribution of HNSCC-derived galanin to the immunomodulatory effects, two approaches were used to reduce galanin: siRNA-mediated silencing in HNSCC cells and antibody-mediated depletion of CM. Both approaches effectively reduced galanin in the CM and enhanced the proliferation of PBMCs, indicating that tumor-secreted galanin plays a relevant role in the inhibition of IL-2/mitogen-induced proliferation of immune cells. Since galanin alone only affected cell survival at 96 h, and as a component of HNSCC-secreted products had no major effect on immune cell survival, we speculate that its anti-proliferative effect observed at 48 h involves direct modulation of the cell cycle and/or interference with IL-2R/TCR signaling. In fact, PBMCs from HNSCC patients showed a reduced proliferation in vitro, suggesting an influence of tumor-derived products. Moreover, a decrease in proliferation has been found to be more pronounced in PBMCs from patients with more advanced HNSCC [10].

As opposed to assessment of immune cell proliferation, in which potent exogenous stimuli (IL-2 and lectin mitogens) were used, other functional outcomes that were assessed (expression/secretion of biologically active mediators, activation of CD3^+^ and CD8^+^ T cells, phenotypic polarization of CD4^+^ T cells) were solely influenced by soluble mediators present in the CM from HNSCC cells. Constitutive expression of galanin was found to be similar in UM-SCC-1 and UM-SCC-22B cells. Surprisingly, we found that differences in the profile of secreted products had a negligible influence on the relative contribution of tumor cell-derived galanin on the immunomodulatory effects of secreted products from each HNSCC cell line. Overall, as a component of the secretome of two HNSCC cell lines, galanin had a suppressive effect on immune cells, reducing activation of T cells and skewing CD4^+^ T cells to anti-inflammatory/pro-tumoral phenotypes (Th2, Tregs). These results are partially consistent with suppressive effects observed with galanin alone. The decrease in Tregs observed with galanin stimulation must be considered in the context of the markers used (CD4/CD25/FOXP3), which may represent a heterogenous population of Tregs [41, 42]. Further characterization of the phenotype (resting/suppressive) will be necessary. Moreover, these results support an important role of tumor-derived galanin in HNSCC-associated immunosuppression. Since depletion of galanin attenuated the immunosuppressive effects of HNSCC-secreted products, future studies should be aimed at investigating the mechanisms underlying the galanin-mediated suppressive effects and whether anti-tumoral immunity is improved by inhibiting/blocking galanin (or GALR2).

In order to test the galanin-mediated immunosuppressive effect on tumor growth, we performed an in vivo assay using the CAM model. Chicken embryos do not develop an immune system till 18 days of development [43, 44], thereby enabling grafting of human HNSCC cells and human PBMCs without inducing cross-species immune responses [24]. Using this system, we found that galanin-primed PBMCs favored tumor growth, indicating that the immunomodulatory effects of galanin are indeed oncogenic.

Additional analysis of TCGA data revealed a greater survival probability for patients with a low galanin expression and high CD8 levels. Since CD8^+^ T cell infiltration in HNSCCs is associated with a better prognosis and treatment response [45, 46], its inverse association with galanin suggests that inhibiting/blocking galanin expression or signaling may be beneficial in future immunotherapy approaches.

In a previous study we showed that HNSCC and secreted products may induce prominent immunosuppression and co-opted immune cells to promote tumor cell proliferation, survival and migration [19]. It is important to note that the goal of this study was to investigate the relative contribution of HNSCC-derived galanin to immunomodulatory effects and not the identification of all HNSCC-secreted factor(s) contributing to tumor growth. Limitations of this study include the use of only two (systemically healthy and male) PBMC donors, of only two HNSCC cell lines with ‘intermediary’ constitutive expression of galanin, the fact that PBMCs do lacking some immune cell types (most notably neutrophils and platelets) and the absence of other cells from the tumor microenvironment (e.g. fibroblasts).

Since galanin secreted by two independent HNSCC cell lines exerted similar immunosuppressive effects, we speculate that the influence of tumor cell-derived galanin on immune cells may be independent of other secreted products. Similar to the intersection between neural and tumor cells, the results of this study suggest that galanin mediates crosstalk between tumor and immune cells via GALR2, which may favor tumor growth and invasion by facilitating immune-escape and co-opting immune cells. Thus, we propose that the galanin/GALR2 axis should be further investigated as a candidate target for rescuing anti-tumoral immunity.

## Supplementary information

ESM 1 (PNG 264 kb)

High resolution image (TIF 59717 kb)

ESM 2 (PNG 611 kb)

High resolution image (TIF 65402 kb)

ESM 3 (DOCX 19 kb)

## Data Availability

All available material and data are presented in the manuscript.**Code availability** Not applicable.

## Abbreviations

7AAD: 7-amino-actinomycin D
ab-Gal: Galanin depleted by Antibody
AKT: RAC-alpha serine/threonine-protein kinase
CAM: Chick Chorioallantoic Membrane
CD25: Cluster of differentiation 25
CD3: Cluster of differentiation 3
CD4: Cluster of differentiation 4
CD69: Cluster of differentiation 69
CD8: Cluster of differentiation 8
CM: Conditioned medium
EGF: Endothelial growth factor
ERK: Extracellular signal-regulated kinases
FGF-2: Fibroblast growth factor 2
FLT3L: FMS-like tyrosine kinase 3 ligand
FOXP3: Forkhead box protein P3
GALR1: Galanin receptor 1
GALR2: Galanin receptor 2
GALR3: Galanin receptor 3
GATA3: Trans-acting T cell-specific transcription factor GATA-3
G-CSF: Granulocyte-colony stimulating factor
GM-CSF: Granulocyte-macrophage colony-stimulating factor
CXCL1: Growth-regulated alpha protein precursor
GTP: Guanosine triphosphate
HNSCC: Head and neck squamous cell carcinomas
IFNγ: Interferon gamma
IgG: Immunoglobulin G
IL-10: Interleukin 10
IL-12A: Interleukin 12A
IL12-P40: Interleukin 12 subunit p40
IL12-P70: Interleukin 12 subunit p70
IL-17A: Interleukin 17A
IL-1RA: Interleukin-1 receptor antagonist
IL-1α: Interleukin 1 alpha
IL-2R: Interleukin-2 receptor
IL-4: Interleukin 4
IL-6: Interleukin 6
M0: Resting macrophage
M1: Classically activated macrophage
M2: Non-classically activated macrophage
MAPK: Mitogen-activated protein kinase
CCL7: Monocyte chemotactic protein-3
MIP-1β: Macrophage inflammatory protein- 1β
NFATC2: Nuclear factor of activated T-cells, cytoplasmic 2
P38: Mitogen-activated protein kinase p38a
PBMCs: Peripheral blood mononuclear cells
PDGF: Platelet-derived growth factor
PDGF-AB/BB: Platelet-derived growth factor AB/BB
CCL5: Regulated upon activation, normal T cell expressed and presumably secreted
RORγt: Related orphan receptor gamma
RT-qPCR: Quantitative reverse transcription polymerase chain reaction
sCD40L: Soluble cluster of differentiation 40 ligand
SDS-PAGE: Sodium dodecyl sulfate–polyacrylamide gel electrophoresis
siGAL: siRNA for galanin
siNT: siRNA non-target
siRNA: Small interfering RNA
T-bet: T-box protein expressed in T cells
TCGA: The cancer genome atlas
TCR: T cell receptor
TGF-β: Transforming growth factor beta
Th1: T helper type 1
Th17: T helper type 17
Th2: T helper type 2
TNF: Tumor necrosis factor
Treg: Regulatory T cells
