# Supplementary figures and images for: Galanin mediates tumor-induced immunosuppression in head and neck squamous cell carcinoma
Source: Cell Oncol (Dordr). 2022 Mar 10;45(2):241–56. doi: 10.1007/s13402-021-00631-y (PMC9050779; doi:10.1007/s13402-021-00631-y)

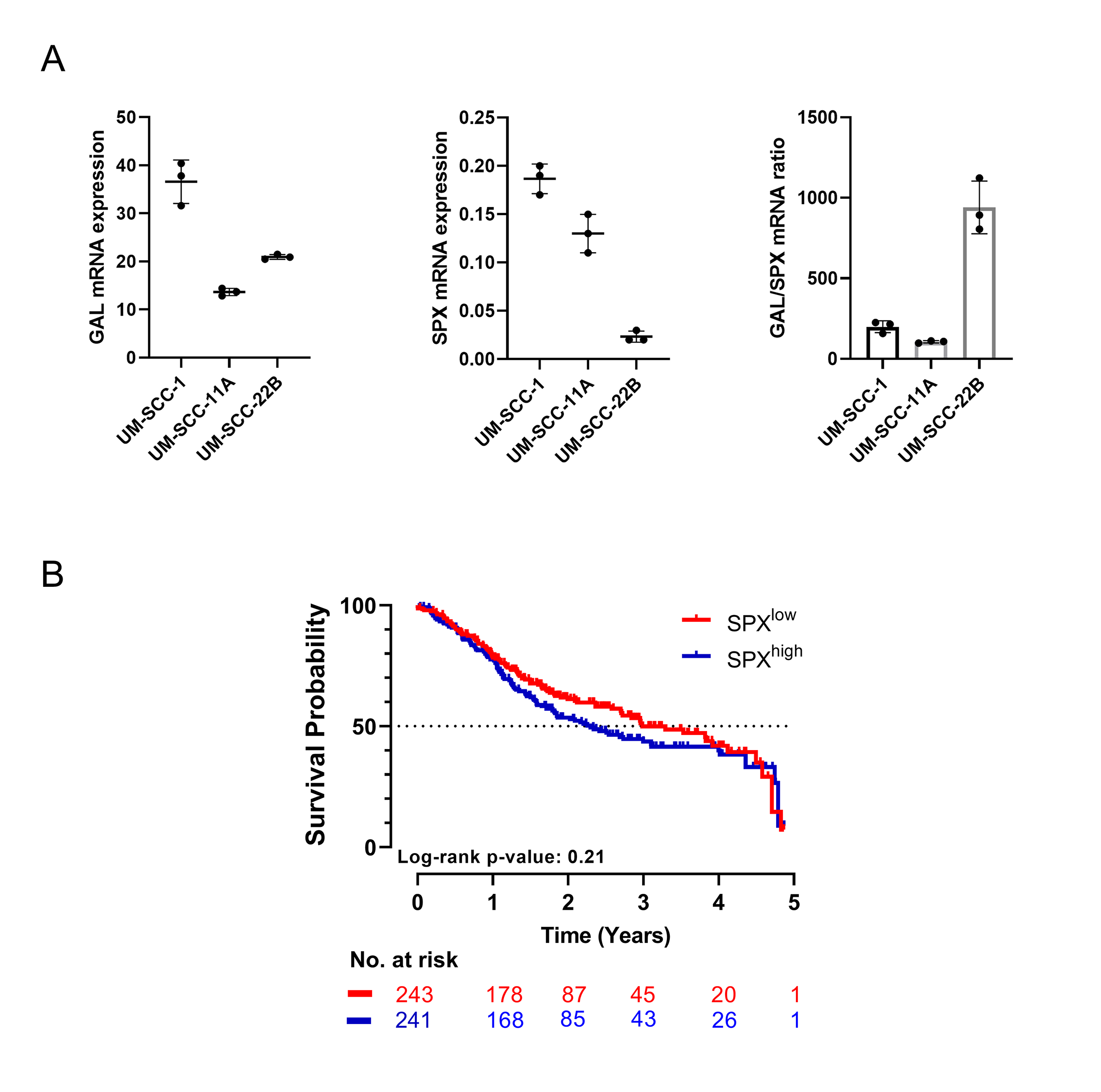

Supplement: Supplementary file 1 — (PNG 264 kb) [file 13402_2021_631_Fig9_ESM.png]

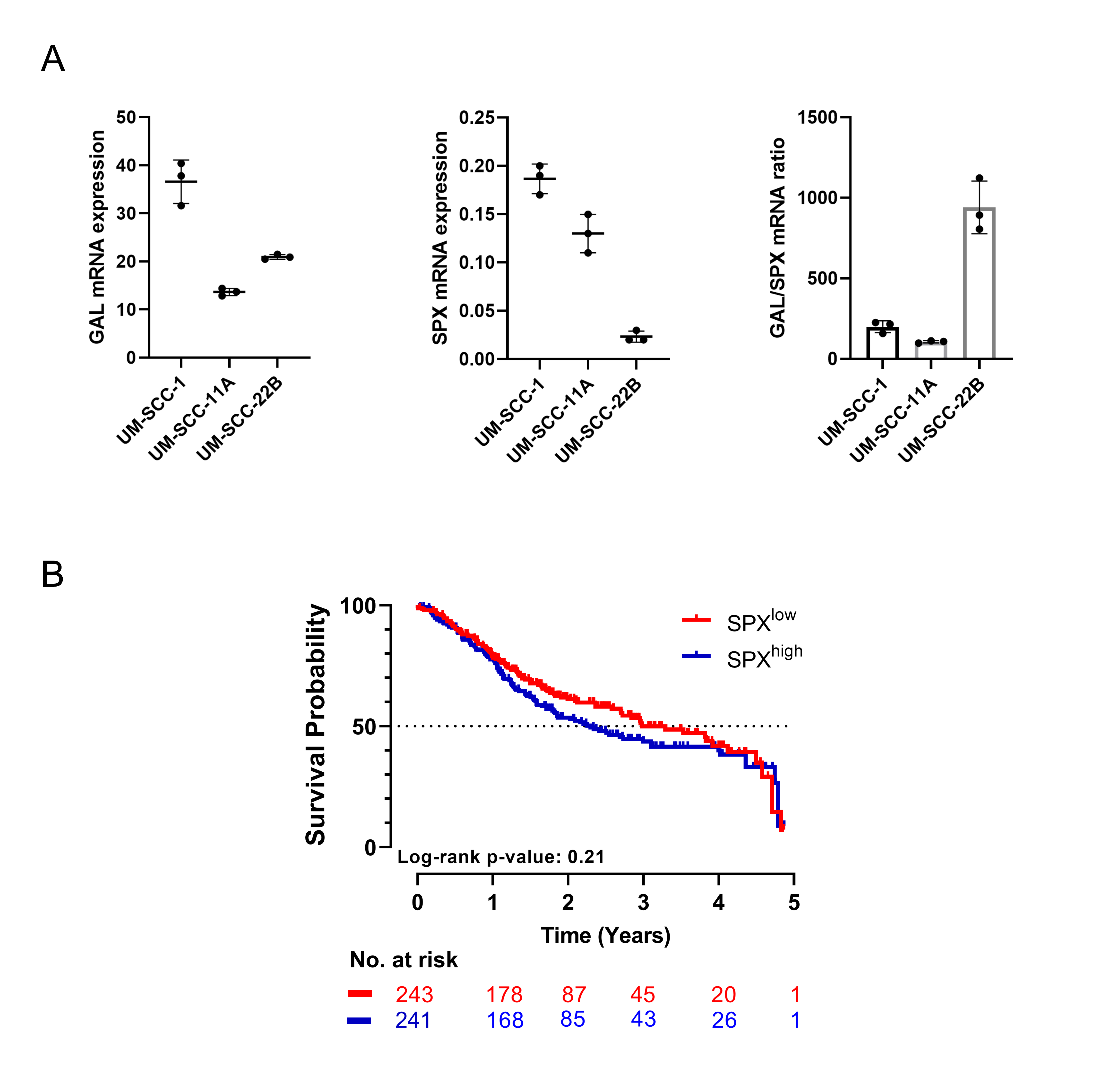

Supplement: Supplementary file 2 — High resolution image (TIF 59717 kb) [file 13402_2021_631_MOESM1_ESM.tif]

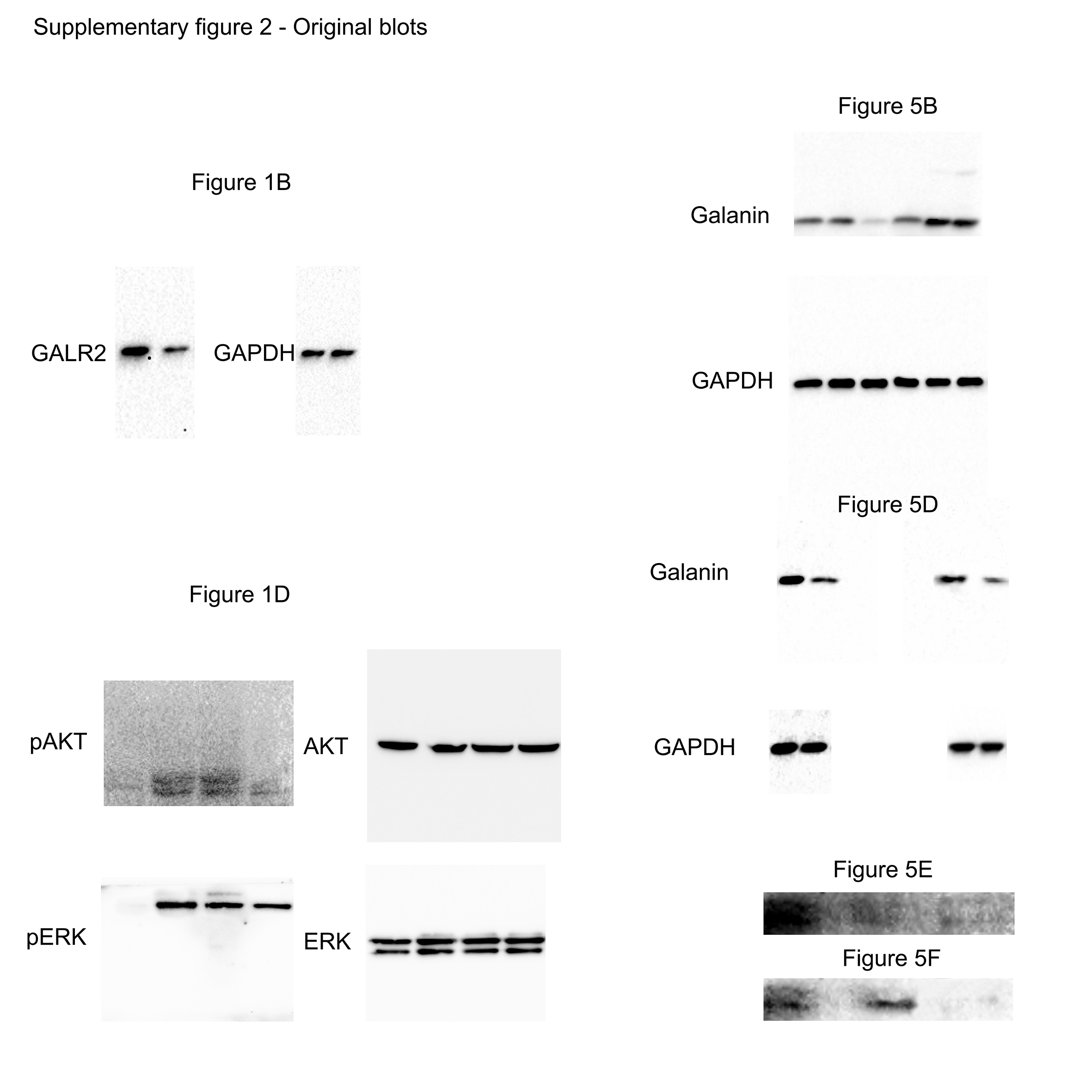

Supplement: Supplementary file 3 — (PNG 611 kb) [file 13402_2021_631_Fig10_ESM.png]

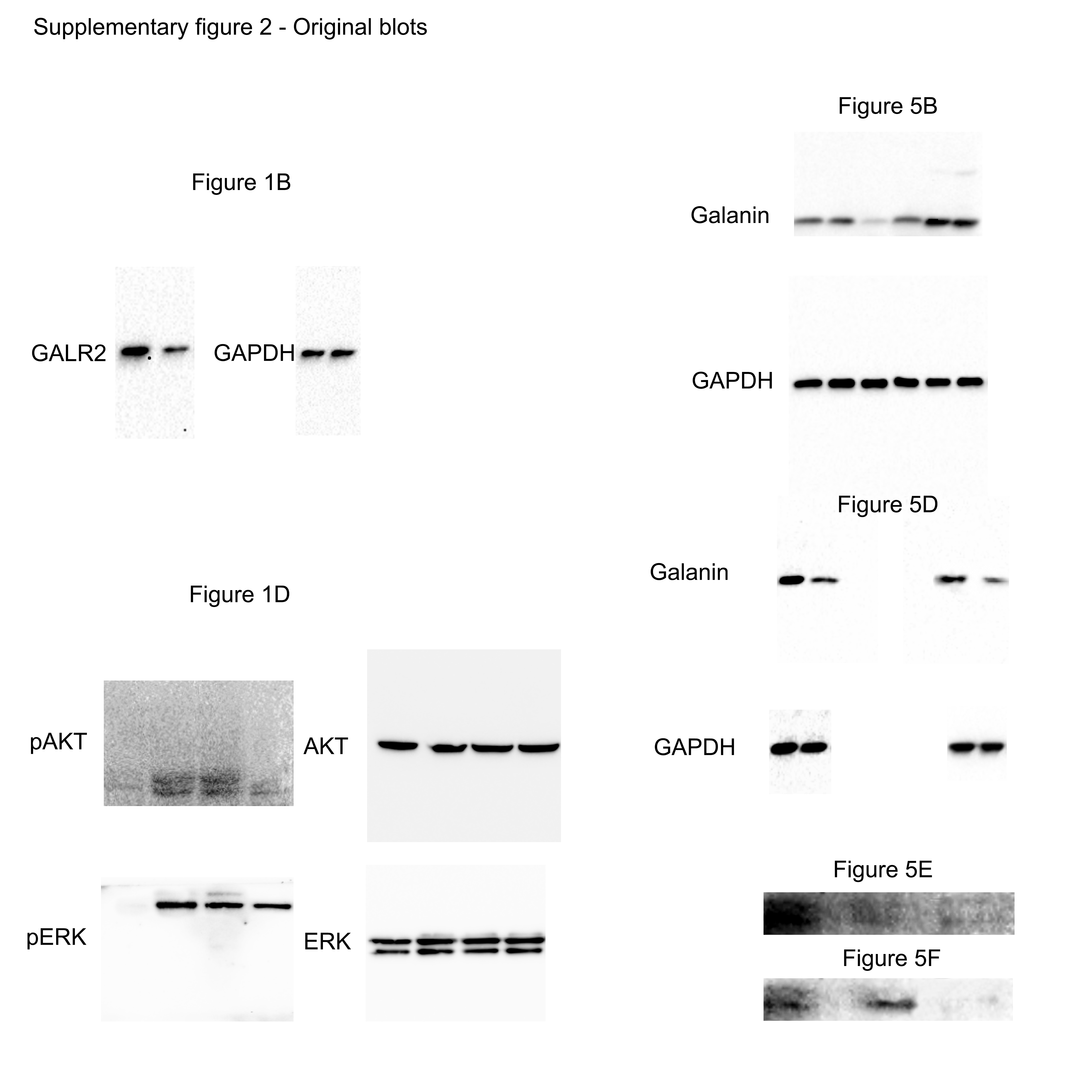

Supplement: Supplementary file 4 — High resolution image (TIF 65402 kb) [file 13402_2021_631_MOESM2_ESM.tif]
