# Supplementary material for: Galanin mediates tumor-induced immunosuppression in head and neck squamous cell carcinoma
Source: Cell Oncol (Dordr). 2022 Mar 10;45(2):241–56. doi: 10.1007/s13402-021-00631-y (PMC9050779; doi:10.1007/s13402-021-00631-y)
Supplement: Supplementary file 5 — (DOCX 19 kb) [file 13402_2021_631_MOESM3_ESM.docx]

**Table S1 - List of reagents**

| **REAGENT or RESOURCE** | **SOURCE** | **Dose/**  **Concentration** | **IDENTIFIER** |
| --- | --- | --- | --- |
| **Antibodies** | | | |
| Rabbit polyclonal anti-galanin | Origene | 1:200 | TA330441 |
| Mouse monoclonal anti-Actin | BD Biosciences | 1:5000 | Cat#612656 |
| Rabbit polyclonal anti-GALR2 | Origene | 1:1000 | Cat# AP01317PU-N |
| Mouse monoclonal anti-GAPDH | Millipore-Sigma | 1:5000 | Cat#MAB374 |
| Rabbit polyclonal anti-p44/42 MAPK | Cell Signaling Technologies | 1:1000 | Cat#9102 |
| Mouse monoclonal anti-phospo-p44/42 MAPK | Cell Signaling Technologies | 1:2000 | Cat#9106 |
| Rabbit polyclonal anti-Akt | Cell Signaling Technologies | 1:1000 | Cat#9272 |
| Rabbit polyclonal anti-phospho-Akt | Cell Signaling Technologies | 1:1000 | Cat#9271 |
| Rabbit Secondary | Cell Signaling Technologies | 1:2000 | Cat#7074 |
| Mouse Secondary | Cell Signaling Technologies | 1:2000 | Cat#7076 |
| **Flow cytometry Ab/Reagents** | | | |
| Annexin V/7-AAD assay | BD Bioscience | - | Cat# 559763 |
| Human BD Fc Block | BD Bioscience | - | Cat#564219 |
| Cytofix/Cytoperm | BD Bioscience | - | Cat#554714 |
| FastImmune CD8-FITC/CD69-PE/CD3-PerCP | BD Bioscience | - | Cat#340367 |
| CD4-PerCP-Cy5.5 | BD Bioscience |  | Cat#341654 |
| IFN-γ-FITC | BD Bioscience |  | Cat#554700 |
| IL-4-PE-Cy7 | BD Bioscience |  | Cat#560672 |
| IL-17A-PE | BD Bioscience |  | Cat#560486 |
| CD4-PerCP-Cy5.5 | BD Bioscience |  | Cat#341654 |
| FOXP3-AlexaFluor | BD Bioscience |  | Cat#488560047 |
| CD25-PE | BD Bioscience |  | Cat#555432 |
| **Biological Samples** | | | |
| PBMC Donor #1 | Cellular Technologies Limited | - | HHu20140804 |
| PBMC Donor #2 | Cellular Technologies Limited | - | HHu20140811 |
| **Chemicals, Peptides and Recombinant Proteins** | | | |
| GALR2 Inhibitor M871 | Tocris | 100 nM | Cat#2698 |
| Galanin | Millipore-Sigma | 10 and 300 nM | Cat#G0278 |
| Recombinant human IL-2 | R&D Systems | 10ng/mL | Cat#P605668 |
| Concavalin A | Millipore-Sigma | 2.5ug/mL | Cat#C5275 |
| Phytohemagglutinin | Millipore-Sigma | 2.5ug/mL | Cat#L1668 |
| Enzyme-free dissociation buffer | Gibco | - | Cat#13151014 |
| Dynabeads™ Human T-Activator CD3/CD28 | Gibco | - | Cat#11131D |
| E. Coli LPS | Millipore-Sigma | 100 ng/ml | Cat#L6529 |
| Trypsin 0.05% | Gibco | - | Cat#25300054 |
| RPMI | Gibco | - | Cat# 72400047 |
| DMEM | Gibco | - | Cat#11995065 |
| Fetal Bovine Serum | Gibco | - | Cat#16140071 |
| Bovine Serum Albumin | Millipore-Sigma | - | Cat#A9418 |
| Cell lysis buffer | Cell Signaling Technologies | - | Cat#9803 |
| TWEEN 20 | Millipore-Sigma | - | Cat#P1379 |
| Dimethyl Sulfoxide | Millipore-Sigma | - | Cat#D8418 |
| Penicillin and Streptomycin | Gibco | - | Cat#15140122 |
| Trypan blue Solution, 0.4% | Gibco | - | Cat# 15250061 |
| Cell Tracker Green CMFDA | Molecular Probes | - | Cat#C7025 |
| Bradford | Bio-Rad | - | Cat#5000006 |
| **Critical Commercial Assays** | | | |
| RNeasy micro kit | Qiagen | - | Cat#74004 |
| SuperScript III cDNA Synthesis kit | Life Technologies | - | Cat#18080051 |
| Human DMBT1 ELISA kit | Biomatik | - | Cat# EKU03679 |
| AMV first-strand cDNA synthesis kit | Invitrogen | - | Cat#E6550 |
| TaqMan Fast Advanced Master Mix | Applied Biosystems | - | Cat#444457 |
| Human Cytokine 41-Plex Panel | Millipore-Sigma | - | Cat# HCYTMAG60PMX41BK |
| MILLIPLEX MAP TGFß Magnetic Bead 3 Plex Kit | Millipore-Sigma | - | Cat# TGFBMAG-64K-03 |
| **Oligonucleotides/Taqman primers** | | | |
| Human Galanin siRNA | Dharmacon | - | Cat# LQ-004139-07 |
| Non-Targeting siRNA | Dharmacon | - | Cat#D-001810-10-05 |
| GAPDH | Applied Biosystems | - | Hs99999905 |
| Galanin | Applied Biosystems | - | Hs00544351_g1 |
| GALR1 | Applied Biosystems | - | Hs00929409_m1 |
| GALR2 | Applied Biosystems | - | Hs00605839_m1 |
| GALR3 | Applied Biosystems | - | Hs00358572_m1 |
| IFNG | Applied Biosystems | - | Hs00989291_m1 |
| IL4 | Applied Biosystems | - | Hs00174122_m1 |
| IL17A | Applied Biosystems | - | Hs00174383_m1 |
| TBX21 | Applied Biosystems | - | Hs00203436_m1 |
| GATA3 | Applied Biosystems | - | Hs00231122_m1 |
| RORC | Applied Biosystems | - | Hs01076122_m1 |
| TRIB2 | Applied Biosystems | - | Hs01120543_m1 |
| ZBTB7B | Applied Biosystems | - | Hs00757087_g1 |
| IL10 | Applied Biosystems | - | Hs0096162_m1 |
| IL12A | Applied Biosystems | - | Hs01073447_m1 |
| **Software and Algorithms** | | | |
| Prism Version 8.0 | GraphPad | - | <https://www.graphpad.com/> |
| SAS v9.4 | SAS Institute | - |  |
| Image J 1.52a | NIH | - | <http://rsbweb.nih.gov/ij/> |
| FACSuite | BD Bioscience | - | - |
| **Other** | | | |
| 50kDa centrifuge filters | Millipore-Sigma | - | Cat#UFC805096 |
| 3kDa centrifuge filters | Millipore-Sigma | - | Cat#UFC800324 |
| Lipofectamine™ RNAiMAX Transfection reagent | Invitrogen | - | Cat#13778030 |
| SuperSignal™ West Pico PLUS Chemiluminescent Substrate | ThermoFisher Scientific | - | Cat#34577 |
| UltraPure DNase/RNase-Free Distilled Water | ThermoFisher Scientific | - | Cat#10977023 |
